# High-multiple spontaneous otoacoustic emissions confirm theory of local tuned oscillators

**DOI:** 10.1186/2193-1801-2-135

**Published:** 2013-03-27

**Authors:** Martin Braun

**Affiliations:** Neuroscience of Music, Gansbyn 14, Värmskog, S-66492 Sweden

**Keywords:** human, cochlea, acoustic frequency interval, frequency spacing order

## Abstract

Understanding the origin of spontaneous otoacoustic emissions (SOAEs) in mammals has been a challenge for more than three decades. Right from the beginning two mutually exclusive concepts were explored. After 30 years this has now resulted in two well established but incompatible theories, the global standing-wave theory and the local oscillator theory. The outcome of this controversy will be important for our understanding of inner ear functions, because local tuned oscillators in the cochlea would indicate the possibility of frequency analysis via local resonance also in mammals. A previously unexploited opportunity to gain further information on this matter lies in the occasional cases of high-multiple SOAEs in human ears, which present a large number of adjacent small frequency intervals. Here, eight healthy ears of four subjects (12 to 32 SOAEs per ear) are compared with individually simulated ears where frequency spacing was random-generated by two different techniques. Further, a group of 1000 ears was simulated presenting a mean of 21.3 SOAEs per ear. The simulations indicate that the typical frequency spacing of human SOAEs may be due to random distribution of emitters along the cochlea plus a graded probability of mutual close-range suppression between adjacent emitters. It was found that the distribution of frequency intervals of SOAEs shows no above-chance probability of multiples of the preferred minimum distance (PMD) between SOAEs and that the size of PMD is related to SOAE density. The variation in size between adjacent small intervals is not significantly different in random-generated than in measured data. These three results are not in agreement with the global standing-wave theory but are in line with the local oscillator theory. In conclusion, the results are consistent with intrinsic tuning of cochlear outer hair cells.

## Background

The global standing-wave theory (GST) and the local oscillator theory (LOT) of SOAE generation have been extensively described and discussed (recently in: Wit and van Dijk 2012). Here the two concepts are only outlined very briefly. The GST, based on concepts of Kemp (1979), Zweig and Shera (1995), and Shera (2003), proposes coherent reflections of basilar membrane (BM) traveling waves between the stapes and points of slight functional irregularities along the cochlear duct, in analogy with the coherent wave reflections in the optical cavity of a laser. Part of the energy of the BM standing wave vibrates the stapes, and via backward middle ear transmission sound is emitted into the ear canal. The standing wave is sustained by energy input from elements of the cochlear amplifier, in particular the outer hair cells (OHC). The LOT, based on concepts of Johannesma (1980), Bialek and Wit (1984), and van Hengel et al. (1996), proposes that the same elements of the cochlear amplifier behave as local oscillators without being coupled to a standing wave. They transmit part of their vibrational energy directly through cochlea and middle ear to the ear canal.

Of the many qualities of SOAEs that the two theories have to account for, perhaps the most complex and demanding one is the observed spacing order of multiple SOAEs in one ear. Schloth (1983) and Dallmayr (1985, 1986) reported a preferred minimum distance (PMD) between spectrally neighboring SOAEs, which appears as outstanding mode in interval histograms. Later studies replicated this result, and Braun (1997) determined on the basis of a pool of 5245 intervals of human SOAEs that the mean PMD amounts to almost exactly 1 semitone (ST) = 1/12 of an octave (recently reviewed in: Wit and van Dijk, 2012).

According to the GST, “the characteristic SOAE spacing can be traced to the value of the wavelength of the traveling wave” (Shera 2003, p. 259). In other words, the GST assumes a general standing wave system that self-stabilizes as the best fit to multiple sites of irregularities. By doing so, it amplifies multiple frequencies simultaneously, leading to multiple SOAEs with a characteristic, wavelength dependent, frequency spacing.

Concerning the LOT, van Hengel et al. (1996) used a mathematical cochlear model to test the effect of frequency distance on mutual interaction of SOAEs. They concluded that “the resulting suppression profile leads to natural minimal distances of effective emissions, without any necessity of additional assumptions about the mechanics of the cochlea” (p. 3570).

Thus, for the LOT the PMD is a short-range effect of mutual interaction of oscillators, whereas for the GST it is a long-range effect of the wavelength of the BM traveling wave. This conflict has the advantage that it can be resolved by experimental data. The simple empirical question is, can the predicted short-range and/or long-range effects be observed in measured SOAE data?

High-multiple SOAEs (>10) in each ear of normal hearing human subjects are occasionally found in large screenings. Indications that SOAE mechanisms might vary according to emission numbers per ear have not been found, and there are no known reasons to expect such a variation. Therefore high-multiple SOAEs can reasonably be regarded as representative for all human SOAEs. Because of their large number of adjacent small SOAE intervals, ears with high-multiple SOAEs provide a unique and previously unexploited opportunity to examine the question of SOAE spacing order, and thus also the question of SOAE generation.

Three groups of results are reported that are not in agreement with the global standing-wave theory but are in line with the local oscillator theory of SOAE generation.

## Results

### Real and simulated data of eight single ears

**Figure 1:**
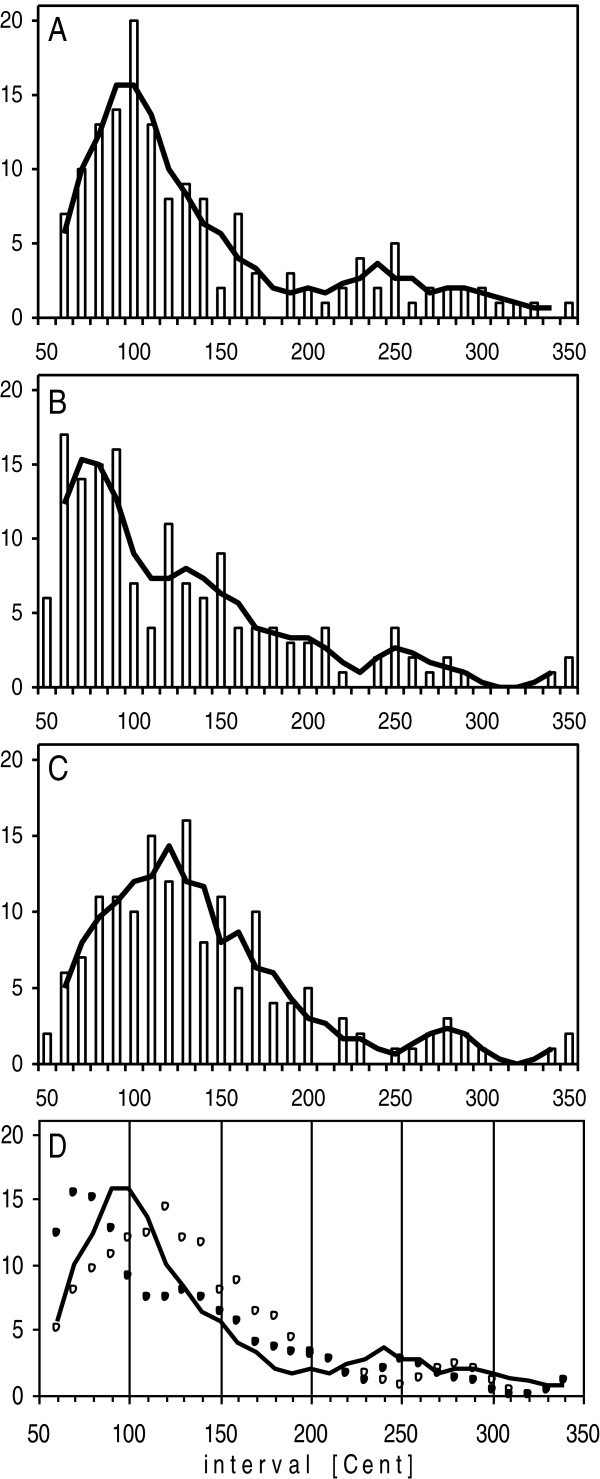
**Distribution of frequency intervals of the 168 SOAEs of the eight ears, displayed on the logarithmic Cent scale [100 Cent = 1 semitone; 12 semitones (ST) = 1 octave].** X-axis: interval size in 10-Cent bins, where each bin is centered around the given scale step. Y-axis: number of intervals per bin. Columns: cases per bin. Lines: 3-point smoothing across bins. **A**. Real data. **B**. Data of first simulation (Sim1). **C**. Data of second simulation (Sim2). **D**. Lines from **A**-**C** in one plot: line: real data; filled circles: Sim1 data; open circles: Sim2 data.

The histogram of frequency intervals between adjacent SOAEs in eight ears (Figure 1A) replicates the well-known mode at 100 Cent = 1 ST. The corresponding histograms of the data from two simulations (Figure 1B, 1C) also each show a single outstanding mode on the small interval side. For the first simulation (Figure 1B) the mode is at 75 Cent = 0.75 ST, for the second simulation (Figure 1C) the mode is at 120 Cent = 1.2 ST.

Considering the single 0.1 ST bins across the three histograms, a conspicuous difference between real and simulated data appears. The real data show a narrow 1-bin mode, whereas the simulations show wider modes of 4 and 3 bins. Comparison of the smoothed data (Figure 1D) shows that the height of the single mode is equally outstanding for the two simulations as for the real data.

Besides the mode at 1 ST there is no further peak in Figure 1A. In particular, it is important to note that at the multiples of 1 ST, i.e. at 2 ST and 3 ST, distribution density is flat. Further, as shown in Figure 1D, at 2 ST and above distribution density both for the real and the simulated data generally is almost flat.

**Figure 2:**
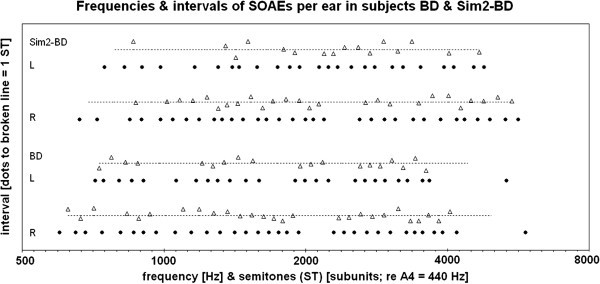
**Frequency spacing of the 57 SOAEs from subject BD. Lower half: real data (BD).** Upper half: data from second simulation (Sim2-BD). R = right ear; L = left ear. X-axis: octave scale, expressed both in frequency [main units, in Hz] and semitones [subunits: 12 semitones = 1 octave]. Y-axis: interval size: distance between each line of SOAE dots and parallel broken line above it is equivalent to PMD = 1 ST. Filled circles (dots): spectral location of SOAEs. Triangles: spectral location and size of all intervals that have a size between 0.5 ST and 1.5 ST.

The lower half of Figure 2 shows the spectral SOAE distribution in the two ears of subject BD. The exact size of all small intervals in the 0.5 to 1.5 ST range is marked by triangles above the respective interval. This technique shows that not only the large intervals (>1.5 ST), but also the small ones, vary considerably from one interval to the next.

Five of the eight ears had five or more cases of pairs of adjacent small intervals in the 0.5 to 1.5 ST range, thus enabling the calculation of an informative mean difference (see first five ears in lines 5 & 6 of Table 1). In each of the five ears the mean difference was close to 0.25 ST. Across all eight ears, based on all individual pairs of adjacent intervals, it was 0.26 ST (SD = 0.19 ST; range 0.00 ST to 0.84 ST). Because PMD ~ 1 ST, it follows that the mean variation between adjacent small intervals was 0.26 of the PMD.

**Table 1:** **Frequency spacing of high-multiple SOAEs in humans**

1	Subjects	BD		JK		DZF7A		MZF13A		Total
2	Ear	R	L	R	L	R	L	R	L	
3	SOAEs	32	25	23	21	23	12	17	15	168
4	Intervals > 0.5 & < 1.5 semitones	27	19	18	11	13	3	6	7	**104**
5	Adjacent intervals from (4)	23	15	14	5	8	0	1	3	**69**
6	Mean difference between adjacent									
	intervals of (5) [in semitones]	0.24	0.28	0.25	0.24	0.26	——	0.16	0.43	**0.26**
7	Standard deviation re (6)	0.20	0.21	0.14	0.11	0.22	——	——	0.41	0.19
1A	**First simulation (Sim1)**	BD-1		JK-1		DZF7A-1		MZF13A-1		
3A	SOAEs	32	25	23	21	23	12	17	15	168
4A	Intervals > 0.5 & < 1.5 semitones	24	17	17	9	14	5	12	10	**108**
5A	Adjacent intervals from (4A)	19	12	14	5	8	2	10	5	**75**
6A	Mean difference between adjacent									
	intervals of (5A) [in semitones]	0.26	0.33	0.32	0.28	0.24	0.36	0.35	0.58	**0.32**
7A	Standard deviation re (6A)	0.16	0.27	0.22	0.20	0.25	0.44	0.31	0.28	0.24
8A	t-test and KS-test of (6) vs (6A)	**NS**	**NS**	**NS**	**NS**	**NS**	——	——	——	**NS**
1B	**Second simulation (Sim2)**	BD-2		JK-2		DZF7A-2		MZF13A-2		
3B	SOAEs	32	25	23	21	23	12	17	15	168
4B	Intervals > 0.5 & < 1.5 semitones	26	16	14	9	10	5	12	10	**102**
5B	Adjacent intervals from (4B)	22	10	8	5	5	2	8	6	**66**
6B	Mean difference between adjacent									
	intervals of (5B) [in semitones]	0.27	0.43	0.26	0.37	0.19	0.36	0.22	0.46	**0.31**
7B	Standard deviation re (6B)	0.21	0.30	0.21	0.23	0.13	0.44	0.20	0.25	0.24
8B	t-test and KS-test of (6) vs (6B)	**NS**	**NS**	**NS**	**NS**	**NS**	——	——	——	**NS**

The upper half of Figure 2 shows the simulated SOAE distribution for subject BD from the second simulation (Sim2). Both real and simulated data show sections of strongly or moderately fluctuating interval size. The statistics of interval variation is shown in Table 1. Statistical tests (lines 8A and 8B of Table 1) show that the difference between real and simulated data never reaches the level of significance (*P* > 0.1 in each test).

### Real and simulated data of groups of ears

**Figure 3:**
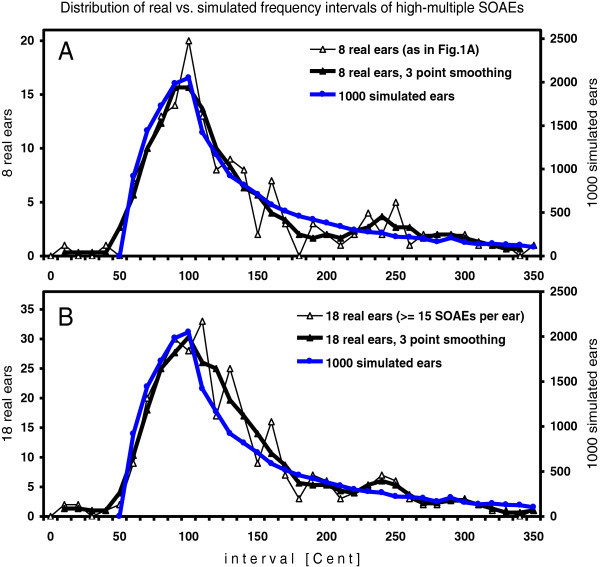
**SOAE interval distribution in real ears and in 1000 simulated ears, displayed as in Figure**1**A.** Intervals of 168 SOAEs from 8 ears (as in Figure 1A) and of 21310 SOAEs from 1000 simulated ears. **B**. Intervals of 346 SOAEs from 18 ears (≥15 SOAEs per ear) and of simulation as in **A**.

The interval distribution of 1000 simulated ears with high-multiple SOAEs in Figure 3A and 3B provides the following information. The distribution is unimodal and heavy-tailed on the right. Due to the large number of simulations, the curve is smooth, and the type of its shape can easily be discerned. The slope left of the peak, which is formed by the linear decrease in existence probability of intervals, shows the shape of a half parabola. The slope right of the peak, which is only formed by random distribution of SOAEs, shows the shape of an exponential decay.

The real data of both ear groups in Figure 3A and 3B show a tendency towards a half parabola left of the peak. Right of the peak, the group data in Figure 3A show a tendency towards an exponential decay, whereas those in Figure 3B show a nearly linear downward slope from 100 to 180 Cent. This difference between the groups of 8 and 18 ears apparently reflects the difference in SOAE density per ear. In the 8-ear group the mean number of emissions per ear was 21, in the 18-ear group it was 19.2. The lower emitter density caused a higher probability of intervals > PMD, as seen in Figure 3B.

**Figure 4:**
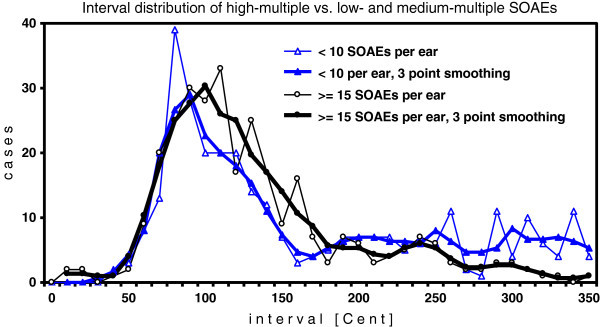
**Interval distribution in ears with high-multiple SOAEs and low- and medium-multiple SOAEs, displayed as in Figures**1**and**3**.** High-multiple SOAE data as from the 346 SOAEs of 18 ears in Figure 3B. Low- and medium-multiple SOAE data based on the 648 SOAEs from 134 ears with <10 SOAEs per ear. The latter group was selected under the condition that the overall mean SOAE frequency matched that of the former group (see Methods section).

The comparison of real data from high-multiple SOAEs with those from low- and medium-multiple SOAEs in Figure 4 shows a conspicuous difference of the location of the peak on the x-axis. Low- and medium-multiple SOAEs show a smaller PMD than high-multiple SOAEs. In the interval range between 50 and 170 Cent the former group has a mean interval of 101.7 Cent (SD = 25.8 Cent), and the latter group has a mean interval of 108.3 Cent (SD = 28.5 Cent). For a test on significance of this difference all intervals in the two data pools had to be considered as independent sample points, because due to the well-known large variation of intervals even within one ear (as in Figure 2 and Table 1) ear-specific or subject-specific clustering of interval size could never be observed. A goodness-of-fit Kolmogorov-Smirnov test (KS-test) of the distribution in 10-Cent bins (Figure 4) showed that neither of the two data sets differed significantly from a normal distribution. An F-test showed that the variances of the two data sets did not differ significantly. Therefore the difference of the mean of the two data sets could be tested by the t-test. The result showed that the difference is significant (*P* < 0.02). Additionally, the two distributions were compared by a non-parametric test, the KS-test. Again the difference was found to be significant (*P* < 0.02).

## Discussion

### Random distribution and close-range suppression

The statistical results in Table 1 show that the simulated spectral distributions of SOAEs in human ears lead to a variation between small adjacent intervals that does not differ significantly from that in the measured data. This result indicates that two factors may be sufficient to cause the observed phenomena: a stochastic occurrence of emission generators along the cochlea plus a graded probability of close-range suppression of an emitter by an adjacent stronger one. The large-scale group simulations (Figure 3) further show that a simple linear increase of suppression probability for intervals from 95 to 55 Cent would be consistent with the measured data from high-multiple SOAEs. The physical basis of graded close-range suppression may lie in the tissue between OHC, such as the reticular lamina and the tectorial membrane. If the amount of tissue between two vibrating OHC decreases, the probability increases that the oscillator with the higher amplitude suppresses its neighbor.

Both the group of 18 ears with high-multiple and the group of 134 ears with low- and medium-multiple SOAEs show the absence of multiples of PMD at 200 and 300 Cent (Figure 4). A periodicity of increased SOAE probability based on the PMD does not appear. Also this observation indicates that the spectrum of emission generators along the cochlea may primarily be stochastic and may secondarily be shaped by close-range mutual suppression.

The size of the PMD is related to the density of SOAEs in an ear (Figure 4). For high-multiple SOAEs it amounts to ca 100 Cent, and for low- and medium-multiple SOAEs it amounts to ca 90 Cent, a difference that is statistically significant (Results, part 2). The physical basis of this difference may lie in the fact that in an ear with high emitter density there is a relevant probability that an emitter has close-range neighbors on both sides, as shown in Figure 2. The summation of destructive vibrational interference from two sides is likely to extend the range of effective suppression. In an ear with many emitters, a single emitter would, on average, need more space to survive suppression from neighbors. This variation of the PMD is therefore consistent with the general indication, derived from the group simulations (Figure 3), that the PMD phenomenon is an effect of graded probability of close-range suppression.

### Consequences for global standing-wave theory (GST)

A central prediction of the GST is that multiple SOAEs in one ear are spaced in relation to the wavelength of a BM standing wave (see Background section). Because multiple SOAEs in one ear usually do not show long chains with similar frequency spacing, an assumption of the GST has been that many SOAEs have “dropped out” from the chain (Shera, 2003). If the “drop-out” hypothesis is correct, spacing statistics should not only show an increased probability of the PMD interval but also of its multiples, such as 2PMD and 3PMD. Talmadge et al. (1993, their Figure Eight) indicated on the basis of data from subject BD that multiples of PMD possibly might have an above-chance probability. Mathematical simulations of the GST showed that distribution modes at multiples of the PMD are an essential and necessary consequence of the GST. If such simulations do not contain a mandatory algorithm for the “drop-out” of SOAEs, effects at PMD multiples can of course not appear (Ku et al. 2008, their Figure Ten-I-d and Ten-II-d). If such a “drop-out” algorithm is applied, e.g., by setting a reasonable threshold of sound level for the detection of SOAEs, effects of PMD multiples become quite conspicuous (Ku et al. 2009, their Figure Thirteen a and Thirteen b). Here, in one GST simulated ear, two cases of 2PMD, three cases of 3PMD, and one case of 6PMD appear.

In an investigation of several other questions, Braun (1997) reported the size distribution of 5245 monaural intervals in the 0–800 Cent range. He observed that multiples of PMD had no above-chance probability and pointed out the consequences for concepts that postulated the opposite. Shera (2003, section VII-E, third paragraph) focused on the fact that the observations of Braun (1997) were based on all possible intervals per ear (all-order intervals), not only on intervals between adjacent SOAEs (first-order intervals). He then argued that summations of small intervals to larger ones would lead to broad peaks in the distribution histogram at the locations of PMD multiples, which therefore possibly would disappear in the noise floor. In other words, he asserted that multiples of PMD would have existed, but were disguised by the technique of analyzing all-order intervals (Shera 2003, section VII-E, last sentence of third paragraph). Thus, Shera was fully aware of the fact that the GST, by definition, predicted a multimodal distribution of SOAE intervals. The present results (Figure 4), which are exclusively based on intervals between adjacent SOAEs (first-order intervals), again show the absence of the predicted multimodal distribution and therefore disprove a central component of the GST.

The finding that the mean variation between adjacent small intervals (0.5 to 1.5 ST) was not significantly different between real and random generated SOAE data (Table 1) also indicates that the spectral spacing of emissions may be stochastic and unrelated to the wavelength of cochlear waves. Clearly, if the regular spacing of the excursion peaks of a BM standing wave influenced the spectral distribution of SOAEs in one ear, SOAE spacing necessarily would show some degree of regularity in the variation between adjacent small intervals. The con-sistency of the measured variation with that in the random generated simulations indicates the absence of such regularity and thus the absence of a relation between SOAE spacing and BM standing waves.

The relation between size of PMD and number of emissions per ear (Figure 4) has not been reported before. Also this finding is inconsistent with the GST. The “drop-out” hypothesis of the GST (see above) can explain that the number of emissions per ear varies according to the number of “drop-outs” from the quasi-periodic chain of emissions that is determined by the wavelength of a BM standing wave. The number of “drop-outs”, however, cannot have a significant causal relation to the wavelength of the standing wave, and thus to the size of the PMD. The wavelength of BM traveling waves is determined by macromechanical parameters of the BM and the cochlea, and the existence or absence of SOAEs cannot significantly be related to these parameters, which is confirmed by the common observation that many healthy and functionally completely normal human ears show no SOAEs at all.

### Consequences for local oscillator theory (LOT)

Early observations that SOAEs interact with each other (Burns et al. 1984) indicated that emission generators, i.e., local oscillators, mutually interact within the cochlea (e.g., van Hengel et al. 1996; Wit and van Dijk 2012). Of particular interest were neighboring oscillators, because the amplitude of radiated waves usually decreases with distance. Because two SOAEs in one ear extremely rarely have an interval below 0.5 ST, it has often been assumed that this fact is due to mutual suppression.

Here, for single ears two types of close-range suppression were simulated, a total suppression beyond a simple low-side limit (Sim1), and a progressive suppression over a low-side range (Sim2). The two simulations have shown that both types of suppression lead to a strong distribution peak slightly above the limitation (Figure 1). The group simulation of 1000 ears showed that a linear increase of suppression probability with decreasing inter-emitter distance almost perfectly mirrored the real data (Figure 3). All simulations taken together indicate that stochastic spacing of emitters plus close-rang suppression alone can explain the PMD phenomenon.

The observation that the PMD slightly varies with SOAE frequency (Methods, part 5) would be compatible both with the GST and the LOT. The anatomy of the cochlear partition varies along the cochlea, which has an effect on the wavelength of traveling waves (relevant for GST) as well as on inter-emitter tissue and thus inter-emitter suppression (relevant for LOT). The relation between emission number per ear and size of PMD (Figure 4), however, is in conflict with the GST (Discussion, part 2), whereas it can be explained by the LOT (Discussion, part 1).

### Relation to other data and to cochlear mechanics

SOAE frequencies are mirrored by frequencies of peaks in any spectrum of evoked OAEs, and also by frequencies of best sensitivity in the micro-spectrum of hearing threshold (Zwicker and Schloth 1984; Talmadge et al. 1998). However, the reverse is not true. Many peaks in the spectra of evoked OAEs and hearing sensitivity are not mirrored by SOAEs. It is still unknown if this discrepancy is due to measurement limitations in recording low-level SOAEs or due to an additional mechanism that is related to evoked OAEs and threshold microstructure but not to SOAEs.

Numerous empirical studies reported evidence that OAE related intracochlear backward transmission toward the stapes occurs via fast compressional sound waves, and not via slow backward traveling waves (Ren 2004; Ren et al. 2006; Ruggero 2004; Siegel et al. 2005, He et al. 2008; He et al. 2010). These results are consistent with the LOT, because the LOT does not require a backward traveling wave, and local oscillator-generated emissions can reach the stapes through the cochlear fluids. However, the intracochlear reflection at the stapes cannot result in standing waves of the basilar membrane, if there is no reverse traveling wave. Therefore, the above referenced data do not support the GST.

Several other statistical properties of human SOAEs are not compatible with the determination of SOAE frequencies by cochlear macromechanics, as suggested by the GST. Above-chance binaural mirroring (Braun 1998), above-chance binaural (but not monaural) frequency ratios that presumably play a role in pitch extraction in the auditory midbrain (Braun 2000), and the bimodal frequency distribution around the two peaks at 1.5 kHz and 3 kHz (Braun 2006), each, and independently, indicate efferent neural influence on the probability of local cochlear oscillations, because alternative mechanisms could neither be found nor be suggested.

Zheng et al. (2011) reported a frequency specific ringing of the BM that indicates local tuning within the organ of Corti. These findings, in combination with the present ones, suggest intrinsic tuning of cochlear outer hair cells (Canlon et al. 1988; Brundin et al. 1989a and Brundin et al. 1989b ; Brundin and Russell, 1994). Additionally, because the barn owl presents SOAEs as high in the frequency range as humans (Taschenberger and Manley, 1997) without motility of hair cell bodies, intrinsic tuning of hair bundle motility (Nam and Fettiplace, 2008) should be considered as a candidate for the origin of SOAEs also in mammals.

### Medical applications

Three decades after their discovery, otoacoustic emissions (OAEs) are today widely used in medical diagnosis. However, uncertainty as to their physiological causes severely impedes further applications. Currently, the main medical application of OAEs is checking the status of hearing. One can expect, however, a much larger potential of OAEs in the context of highly complex disorders, such as tinnitus (Geven et al. 2012) or Ménière’s disease (Avan et al. 2011). For example, diagnostic tests on tinnitus or Ménière’s disease that use OAEs in combination with macromechanical manipulations of the cochlea (Nubel et al. 1995), such as low-frequency biasing of BM position (Scholz et al. 1999), are likely to benefit from knowledge of OAE physiology.

## Conclusions

The distribution of frequency intervals of human spontaneous otoacoustic emissions (SOAEs) shows no above-chance probability of multiples of the preferred minimum distance (PMD) between SOAEs. The size of PMD is related to SOAE density. The variation in size between adjacent small intervals is not significantly different in random-generated than in measured data. Each of these three results is contrary to the predictions of the global standing-wave theory (GST) but in agreement with the local oscillator theory (LOT) of SOAE generation. Overall, the results are consistent with intrinsic tuning of cochlear outer hair cells (OHC) as a key functional element in the frequency analysis of the inner ear.

## Methods

### Primary data set

There are two preconditions for the collection of a relevant number of high-multiple SOAEs: the screening of many subjects (>100), and the best possible techniques for recording and signal analysis. Several survey studies were carried out in the early 1990s, when the principal aim was to establish prevalence conditions of SOAEs in humans. The ones that collected the largest numbers of SOAEs were those of Russell (1992) and Talmadge et al. (1993). These authors applied similar advanced techniques, which is reflected by the similar statistical results that they reported. In both studies the best available methods of acoustic recording and data extraction, which have not further developed since then, were applied. The raw data from these two surveys were re-used in many later investigations (for a recent example: Wit and van Dijk 2012, Figure Ten a-d and acknowledgments). Here, from each of these two studies the data from the two subjects presenting the highest numbers of SOAEs were investigated by newly developed techniques. The four subjects, BD, JK, DZF7A, and MZF13A were all adult females, healthy, and normal hearing. At their ages of 34, 20, 21, and 21 they presented 57, 44, 35, and 32 SOAEs, respectively.

For each of the eight ears, all frequency intervals between adjacent SOAEs were calculated into values of the logarithmic Cent scale [100 Cent = 1 semitone (ST); 12 ST = 1 octave]. Then the intervals of the 168 SOAEs of the eight ears were pooled and their size distribution was calculated for bins of 10 Cent = 0.1 ST and displayed in a histogram.

### First simulation of eight single ears

The possible effects of mutual suppression of adjacent SOAEs on spacing order were investigated by using two different simulations. The first simulation applied a simple low-side limit of interval size for each ear, i.e., intervals that were smaller than a given value were excluded. The second simulation applied a progressive range of existence probability on the low-end side of interval size, i.e., the probability of small intervals decreased progressively toward the given smallest interval. Both simulations had in common that each of the eight real SOAE distributions per ear was mirrored by an individually simulated, random-generated distribution.

For the simulations the RANDBETWEEN function of the software package Microsoft Excel (version 9.0.3821 SR-1 from 2000) was used. This function generates, for each round of application, one random selected integer from within a given range between two integers, such that each integer within the range occurs with equal probability. The randomness is algorithm based and its quality is generally considered as fully sufficient for medium size simulations as the present ones. Indications to the contrary have not become known over the period of twelve years that this software package has been in use worldwide (internet scans). Here, the function was used to generate simulated SOAE frequencies, randomly with equal occurrence probability, from within the SOAE frequency range of the real ear. For example, for the simulation of the right ear of subject BD, 32 SOAE frequencies were generated from within the range of 629 Hz to 6140 Hz.

In the first simulation, the empirically observed general low-side limit of ~ 0.5 ST for intervals between adjacent SOAEs (Braun 1997) was simulated as follows. First, the smallest interval in the real ear was determined, e.g., 0.67 ST in the right ear of subject BD. Second, from this value a low-side limit for the simulated intervals of this ear was derived by using the nearest low-side multiple of 0.10 ST, such as 0.50 ST, 0.60 ST, 0.70 ST, etc., as the exclusion criterion. Thus for this ear the low-side limit was 0.60 ST, leading to a rejection of intervals < 0.61 ST. Third, after random generation, the higher SOAE frequency of all pairs whose interval fell into the rejection zone, i.e. was too small, was deleted and replaced by a new random-generated SOAE frequency. Fourth, the replacement procedure was repeated until the low-side criterion was satisfied for all intervals of this ear, e.g., no interval was < 0.61 ST.

For ears BD-R, JK-L, DZF7A-R, and MZF13A-R the exclusion criterion was < 0.61 ST. For the other four ears it was < 0.51 ST. When determining the exclusion criteria, the extremely small interval of 0.11 ST in MZF13A-R was neglected as an extreme outlier and the equally untypical interval of 0.36 ST in DZF7A-L was taken to justify the lower of the two typical limits, i.e. < 0.51 ST. Separately for the two rounds of simulations, the interval distribution of the 168 simulated SOAEs of the eight simulated ears was analyzed in the same way as for real SOAEs.

### Second simulation of eight single ears

In the second simulation, the results from the first simulation were reprocessed in order to take into account the gradual decrease of intervals between 0.9 ST and 0.5 ST in the real data. The progressive decrease of intervals towards the smallest one was simulated by using several limits at steps of 0.1 ST, with each limit mirroring the real data. For example, in the right ear of subject BD the four 0.1 ST bins between 0.5 ST and 0.9 ST show 0, 4, 0, and 5 cases, respectively. These numbers were taken as maximum in the corresponding bins in the second simulation, and the same deletion and replacement procedure as in the first simulation was applied until all maximum-per-bin conditions were satisfied. It should be noted that the number of simulated intervals could in the end be below the given maximum of some bins, because each deletion and replacement of a random-generated SOAE frequency leads to a reordering of the complete chain of SOAE frequencies per ear. Further, it was not possible to add an interval to a bin, because random generation does not permit this. Therefore, the data of the second simulation unavoidably showed slightly less small intervals below 0.9 ST than the real data.

Possible long-range effects in SOAE spacing order were investigated by computing the size variation between adjacent small intervals. Because PMD amounts to almost exactly 1 ST with a range from ~ 0.5 ST to ~ 1.5 ST at the base of the distribution mode (Braun 1993 and Braun 1997), all intervals between 0.5 and 1.5 ST entered into the analysis. The interval size variation was analyzed statistically, separately per ear and also for the total of all eight ears, by comparing real and simulated data. Goodness-of-fit Kolmogorov-Smirnov tests (KS-test) showed that the data sets are consistent with a normal distribution and F-tests showed that the variances do not differ significantly. Therefore application of the t-test was appropriate. Additionally, the data set of the total of the eight ears was compared with each the two data sets of the total of the eight simulated ears by a non-parametric test, the KS-test.

### Simulation of a group of 1000 ears

Additionally to the simulation of single ears, the effect of decreasing existence probability of small intervals with decreasing interval size was also simulated for high-multiple SOAEs in general. Van Hengel and Maat (1993, their Figure Four) showed in a simple mechanical model that the probability of an emission being suppressed by an adjacent stronger one increases with decrease of interval size. Here, 1000 ears with a possible maximum of 32 SOAEs each, in the frequency range of the right ear of subject BD (629 Hz to 6140 Hz), were simulated by random generation (as described above) and a simple linear decrease of existence probability of intervals from 100 to 50 Cent.

The existence probability decreased in five steps of 20%. Above 95 Cent intervals had an existence probability of 100%, for the range of 95 to 86 Cent it was 80%, for 85 to 76 Cent 60%, for 75 to 66 Cent 40%, for 65 to 56 Cent 20%, and below 56 Cent 0%. The 1000 ears were simulated in five blocks of 200. In the first block no intervals < 96 Cent were permitted, in the second no < 86, in the third no < 76, in the fourth no < 66, and in the fifth no < 56 Cent. For each SOAE of each ear a separate random generation was carried out. First, 32 SOAEs in the given range, 629 Hz to 6140 Hz, were generated. Second, the lowest number of the 32 was selected as the first SOAE, and all other numbers were discarded. Third, 31 SOAEs in an adapted new range, between the first SOAE plus permitted minimum distance (in Hz) and 6140 Hz, were generated. Fourth, the lowest number of the 31 was selected as second SOAE, and again all other numbers were discarded. Fifth and following, adaptation of range, random generation, and selection of lowest number was repeated until the adapted range was smaller than the permitted minimum distance, such that no further random generation was possible.

Due to the random character of the procedure, the resulting number of SOAEs per ear always was below 32. Across the 1000 simulated ears the mean number of SOAEs per ear was 21.310 and the total number of SOAEs was 21310. Therefore the simulation provided no optimum representation of the right ear of subject BD. But it provided an adequate representation of the group of eight ears with high-multiple SOAEs, as analyzed above, because the mean number of SOAEs per ear in this group was 21. The simulated ears presented 20310 intervals between adjacent SOAEs, which were plotted in a distribution diagram in contrast to the group data of the eight real ears as analyzed above. In a further diagram they were also plotted in contrast to the group data of the 18 ears from the two surveys of Russell (1992) and Talmadge et al. (1993) that presented ≥15 SOAEs per ear with a group mean of 19.2 SOAEs per ear.

### Empirical data of high-multiple vs. low- and medium-multiple SOAEs

For a comparison of the data from high-multiple SOAEs with those from low- and medium-multiple SOAEs, all ears from the two surveys of Russell (1992) and Talmadge et al. (1993) that presented <10 SOAEs per ear were considered. Because van Hengel et al. (1996, their Figure Four) and Shera (2003, his Figure Three), by using data from Talmadge et al. (1993), had shown that the PMD, as an octave fraction, slightly decreased with the increase of the mean frequency of SOAE pairs, it was necessary to compare data sets with a similar overall mean of SOAE frequency. For the above-described group with ≥15 SOAEs per ear the overall mean was 2144 Hz. For the above described group with <10 SOAEs per ear it was 2320 Hz. The latter group therefore was reduced in order to obtain two groups with matching overall mean SOAE frequency. For this purpose the mean SOAE frequency for each single ear in the latter group was determined. Then the ear with the highest mean was iteratively deleted from the group until the group’s overall mean was within ± 0.005 of the mean of the other group. After completion of the procedure the overall mean of the reduced group with <10 SOAEs per ear was 2151 Hz, and the reduced group’s 134 ears presented 648 SOAEs, the mean being 4.8 SOAEs per ear.

## Abbreviations

BM: Basilar membrane
GST: Global standing-wave theory
LOT: Local oscillator theory
OAE: Otoacoustic emission
OHC: Outer hair cell
PMD: Preferred minimum distance
SOAE: Spontaneous otoacoustic emission
ST: Semitone.
